# Mass drug administrations with dihydroartemisinin-piperaquine and single low dose primaquine to eliminate *Plasmodium falciparum* have only a transient impact on *Plasmodium vivax*: Findings from randomised controlled trials

**DOI:** 10.1371/journal.pone.0228190

**Published:** 2020-02-05

**Authors:** Koukeo Phommasone, Frank van Leth, Thomas J. Peto, Jordi Landier, Thuy-Nhien Nguyen, Rupam Tripura, Tiengkham Pongvongsa, Khin Maung Lwin, Ladda Kajeechiwa, May Myo Thwin, Daniel M. Parker, Jacher Wiladphaingern, Suphak Nosten, Stephane Proux, Chea Nguon, Chan Davoeung, Huy Rekol, Bipin Adhikari, Cholrawee Promnarate, Kesinee Chotivanich, Borimas Hanboonkunupakarn, Podjanee Jittmala, Phaik Yeong Cheah, Mehul Dhorda, Mallika Imwong, Mavuto Mukaka, Pimnara Peerawaranun, Sasithon Pukrittayakamee, Paul N. Newton, Guy E. Thwaites, Nicholas P. J. Day, Mayfong Mayxay, Tran Tinh Hien, Francois H. Nosten, Frank Cobelens, Arjen M. Dondorp, Nicholas J. White, Lorenz von Seidlein

**Affiliations:** 1 Lao-Oxford-Mahosot Hospital-Wellcome Trust Research Unit (LOMWRU), Microbiology Laboratory, Mahosot Hospital, Vientiane, Lao People’s Democratic Republic; 2 Department of Global Health, Amsterdam University Medical Centers, Location Academic Medical Center, Amsterdam, Netherlands; 3 Amsterdam Institute for Global Health & Development, Amsterdam, Netherlands; 4 Mahidol Oxford Tropical Medicine Research Unit, Faculty of Tropical Medicine, Mahidol University, Bangkok, Thailand; 5 Nuffield Department of Medicine, Centre for Tropical Medicine and Global Health, University of Oxford, Oxford, England, United Kingdom; 6 Shoklo Malaria Research Unit, Mahidol-Oxford Tropical Medicine Research Unit, Faculty of Tropical Medicine, Mahidol University, Mae Sot, Thailand; 7 Institut de Recherche pour le Développement (IRD), Institut national de la santé et de la recherche médical (INSERM), Aix-Marseille Université · SESSTIM, Marseille, France; 8 Oxford University Clinical Research Unit, Wellcome Trust Major Oversea Programme, Ho Chi Minh City, Vietnam; 9 Savannakhet Provincial Health Department, Savannakhet Province, Lao People’s Demographic Republic; 10 Department of Clinical Tropical Medicine, Faculty of Tropical Medicine, Mahidol University, Bangkok, Thailand; 11 Department of Population Health and Disease Prevention, University of California, Irvine, California, United States of America; 12 National Center for Parasitology, Entomology and Malaria Control, Phnom Penh, Cambodia; 13 Provincial Health Department, Battambang, Cambodia; 14 Worldwide Antimalarial Resistance Network (WWARN) Asia Regional Centre, Mahidol University, Bangkok, Thailand; 15 Department of Molecular Tropical Medicine and Genetics, Faculty of Tropical Medicine, Mahidol University, Bangkok, Thailand; 16 The Royal Society of Thailand, Dusit, Bangkok, Thailand; 17 Institute of Research and Education Development, University of Health Sciences, Vientiane, Lao People’s Demographic Republic; George Washington University School of Medicine and Health Sciences, UNITED STATES

## Abstract

**Background:**

Mass administrations of antimalarial drugs (MDA) have reduced the incidence and prevalence of *P*. *falciparum* infections in a trial in the Greater Mekong Subregion. Here we assess the impact of the MDA on *P*. *vivax* infections.

**Methods:**

Between May 2013 and July 2017, four villages in each Myanmar, Vietnam, Cambodia and Lao PDR were selected based on high prevalence of *P*. *falciparum* infections. Eight of the 16 villages were randomly assigned to receive MDA consisting of three-monthly rounds of three-day courses of dihydroartemisinin-piperaquine and, except in Cambodia, a single low-dose of primaquine. Cross-sectional surveys were conducted at quarterly intervals to detect Plasmodium infections using ultrasensitive qPCR. The difference in the cumulative incidence between the groups was assessed through a discrete time survival approach, the difference in prevalence through a difference-in-difference analysis, and the difference in the number of participants with a recurrence of *P*. *vivax* infection through a mixed-effect logistic regression.

**Results:**

3,790 (86%) residents in the intervention villages participated in at least one MDA round, of whom 2,520 (57%) participated in three rounds. The prevalence of *P*. *vivax* infections fell from 9.31% to 0.89% at month 3 but rebounded by six months to 5.81%. There was no evidence that the intervention reduced the cumulative incidence of *P*.*vivax* infections (95% confidence interval [CI] Odds ratio (OR): 0.29 to 1.36). Similarly, there was no evidence of MDA related reduction in the number of participants with at least one recurrent infection (OR: 0.34; 95% CI: 0.08 to 1.42).

**Conclusion:**

MDA with schizontocidal drugs had a lasting effect on *P*. *falciparum* infections but only a transient effect on the prevalence of *P*. *vivax* infections. Radical cure with an 8-aminoquinoline will be needed for the rapid elimination of vivax malaria.

## Introduction

Malaria transmission has fallen in the Greater Mekong Subregion (GMS) since the early 2000s. *P*. *falciparum* infections have declined at a faster rate than *P*. *vivax* infections resulting in the current dominance of *P*. *vivax* in most parts of the GMS [1]. The steady decline in *P*. *falciparum* transmission has stalled over the last years coinciding with the emergence and spread of multidrug-resistant *P*. *falciparum* [1]. The spread of resistance is cause for international concern, as the earlier spread of multidrug-resistant *P*. *falciparum* from Asia to sub-Saharan Africa, had devastating public health consequences [2]. Particularly in areas of low malaria transmission, mass antimalarial drug administrations (MDA) have the potential to accelerate the elimination of malaria. The MDA described here resulted in a substantial reduction of *P*. *falciparum* infections over a 12-month period [3].

*P*. *vivax*, in contrast to *P*. *falciparum*, has persistent liver stages (hypnozoites) which cause relapse. Relapse patterns vary geographically. In southeast Asia, *P*. *vivax* tends to have short relapse intervals [4]. Repeated relapses continue the transmission of vivax malaria and impact adversely on health and quality of life. Multiple vivax relapses are a considerable burden in children affecting growth, development and education [5]. Clearing hypnozoites requires a radical cure with a course of 8-aminoquinolines, but this class of drugs is underutilized related to the risk of haemolysis in individuals with glucose-6 phosphate-dehydrogenase (G6PD) deficiency.

Here we report a post hoc analysis of the concomitant effect of a MDA with dihydroartemisinin-piperaquine (DP) plus a single low dose primaquine (SLDPQ) on the prevalence, incidence, and frequency of recurrence of *P*. *vivax* infections in the GMS. SLDPQ is a potent *P*. *falciparum* gametocytocide but has neither measurable activity against *P*. *vivax* hypnozoites nor does it cause clinically relevant haemolysis in G6PD deficient people.

## Methodology

### Trial design

Pre-trial *Plasmodium* prevalence surveys using ultrasensitive PCR (uPCR) in 62 villages in the four countries identified the villages with the highest *P*. *falciparum* prevalence. In each country, four villages with high *P*. *falciparum* prevalence and all-year access were included in the study. Village pairs of similar size, access and *P*. *falciparum* prevalence were randomly allocated to intervention or control using a computer-generated random sequence, except in Myanmar where the drug allocation was decided by flipping a coin. Participants were enrolled by specially trained field staff with the help of local health workers. Participants and field staff were not blinded to the treatment allocation. However, laboratory personnel, who performed the uPCR off-site, did not know which samples were from the intervention or control villages, thereby reducing potential bias.

### Study site

**Fig 1:**
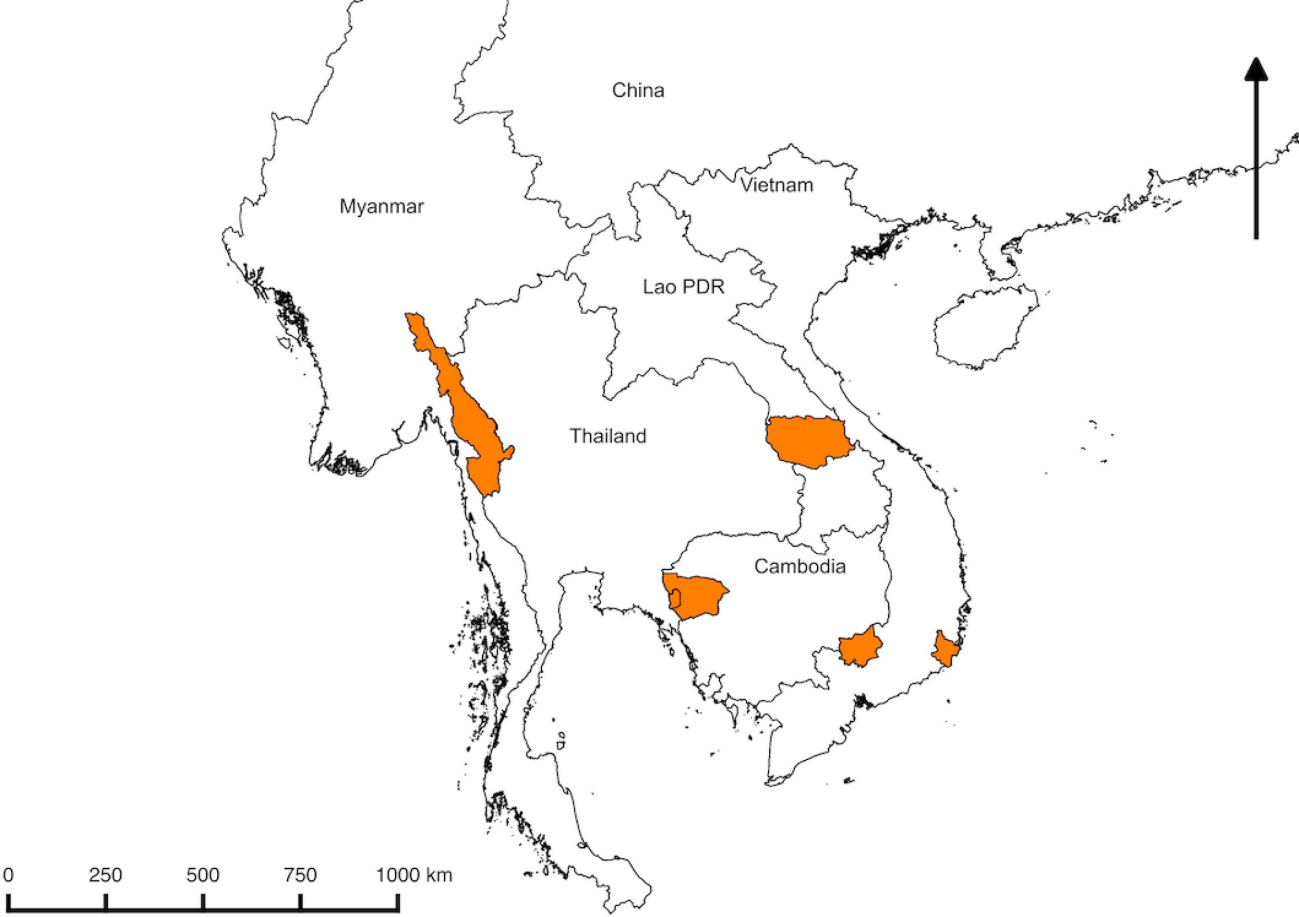
Map of the Greater Mekong Subregion. The areas highlighted in orange are study sites: Kayin (Karen) state, Myanmar; Battambang province, Cambodia; Savannakhet Province, Lao PDR; Binh Phuoc and Ninh Thuan province, Vietnam.

The study was conducted in rural Eastern Kayin (Karen) State of Myanmar; in two provinces of Vietnam (Binh Phuoc and Ninh Thuan provinces: the former is in the Southeast region of the country and shares a border with Cambodia, while the latter embraces the South Central Coast of Vietnam); in Samlout District of Battambang province, Western Cambodia which shares a border with Thailand), and in Nong Districts of Savannakhet province, southern Lao PDR which shares a border with Vietnam. The location of the study sites is shown in Fig 1. Detailed descriptions of the study sites have been published recently [6–9].

### Participants

Every person in the selected villages was eligible to be included in the surveys if they gave informed consent, were older than 6 months, were not ill at the time of the survey, and had no known allergy to the study drugs. All pregnant women were excluded from the MDA in Cambodia and Lao PDR, while in Myanmar and Vietnam only pregnant women in first trimester were excluded. Breastfeeding women were invited to participate but did not receive SLDPQ. Participants who experienced serious adverse events potentially related to study drugs or could not tolerate the study drugs were withdrawn from the next MDA round but remained in the study.

### Intervention

The intervention consisted of three rounds of DP on three consecutive days. One SLDPQ which clears *P*. *falciparum* gametocytes but has no effect on hypnozoites, was added to each round of antimalarials. The treatment regimen was given at the start and then one and two months after start of the study. SLDPQ was not administered in Cambodia for regulatory reasons. A weight-based regimen containing a total dose of approximately 7 mg/kg dihydroartemisinin and 55 mg/kg piperaquine phosphate was used (Eurartesim^®^, Sigma Tau, Italy or D-Artepp^®^, Guilin Pharmaceutical Co, Guilin, People’s Republic of China), while 0.25 mg base/kg PQ was given as a single dose (Government Pharmaceutical Organization, Thailand). The intervention was extended to the initial control villages after 12 months of follow-up, except in Myanmar where the control villages received the intervention after nine months for operational reasons (difficult access during rainy season).

### Data collection and laboratory methods

The study started in Myanmar in May 2013, followed by Vietnam in November 2013, Cambodia in July 2015 and Lao PDR in April 2016. Data were collected through community-wide surveys at baseline, and then every three months. Surveys were preceded by a community census to re-enumerate the study population and to provide intensive community engagement in order to improve high coverage [10–14]. Information collected included demographics, bednet use, duration of stay in the forest, and adverse events. Malaria prevalence at each survey was assessed by quantitative uPCR. 3 mL of blood was taken from participants older than 4 years old, and 0.5 mL from younger children. The blood samples were stored in cool boxes in the field for about 5–6 hours before being separated in the local laboratories and then kept in -20°C freezers. Samples from Myanmar, Cambodia, and Lao PDR were shipped on dried ice to the Mahidol-Oxford Research Unit (MORU) in Bangkok, Thailand for uPCR while samples collected in Vietnam were sent to the Oxford University Clinical Research Unit (OUCRU) in Ho Chi Minh City, Vietnam. DNA was extracted from thawed packed red blood cells using automated DNA extraction, dried in a centrifugal vacuum concentrator, and used as template for PCR detection and quantification of Plasmodium. The methodology and performance characteristics of the quantitative ultrasensitive PCR (uPCR) have been described previously [15].

### Sample size

The sample size described here was for assessing the impact of MDA on the *P*. *falciparum* elimination. Four-village clusters per country, was chosen mainly for operational and practical reasons. It is acknowledged that although the intracluster correlation coefficients (ICCs) were generally very low in this region, there remains a problem of generalisability of findings when such few numbers of clusters are used [16]. A formal sample size calculation suggested that 16 villages (8 villages in each arm) with a minimum of at least 152 individuals in each village recruited and followed up would provide 80% power to detect a 95% fall in prevalence from a 10% initial prevalence, independently powered by country. This calculation assumes the highest ICC of 0.082 for *P*. *falciparum* baseline prevalence in the region that was observed in Laos as a post-study analysis of the ICCs [16].

### Outcomes

The aim of our post hoc analysis was to describe the impact of the MDA on the prevalence of *P*. *vivax* infections at each quarterly survey, cumulative incidence of *P*. *vivax* parasitaemias derived from uPCR results of the quarterly surveys, and frequency of recurrent *P*. *vivax* infections. Recurrent infections after treatment of the blood stage infection can be due to recrudescence of a persistent infection, relapse due to activation of hypnozoites, or reinfection following a new infectious mosquito bite.

### Statistical analysis

The changes of prevalence of *P*. *vivax* infection at the separate surveys were assessed by a difference-in-difference (DiD) approach for which we fitted a logistic model that incorporated effect modification between the intervention groups and the time period of the survey. We assessed these differences between baseline and each follow-up time point (month 3, 6, 9, and 12) with the exception of month 12 data from Myanmar (where for logistic reasons the cross-over MDA in control villages had to be conducted on M9 instead of M12). None of the parameters in the DiD analysis was constrained to zero. The cumulative uPCR-derived incidence of *P*. *vivax* parasitaemias over the 12-month period was assessed using a survival analysis approach in which participants were censored when diagnosed, lost to follow-up, end of the observation period, which ever came first. The effect of the intervention on the incidence of *P*. *vivax* parasitaemias was assessed through a discrete time survival approach using a pooled mixed effects logistic regression analysis incorporating random effects for country and village. A participant was considered to have a recurrent *P*. *vivax* infection if there were two or more episodes with a positive uPCR result.

As consecutive positive uPCR tests could reflect continuous blood stage infection or frequent relapses, we defined an episode in two ways. In the first approach, each positive uPCR test was considered as a separate episode (i.e. relapse or reinfection). In the second approach, consecutive positive uPCR results were considered to belong to the same continuous infection (S1 Table). In two additional sensitivity analyses we considered a missing uPCR test results either as positive or negative (S2–S7 Tables). We assessed the impact of MDA on the recurrence of *P*. *vivax* parasitaemias through mixed-effects logistic regression with random effects for country and village.

All analyses used an individual weight that corrected for panel attrition during the observation period, and non-response at the separate surveys [17]. The panel-attrition weight is the inverse of the probability of participating in all cross-sectional surveys given personal characteristics and characteristics present during follow-up. The cross-sectional non-response weight is the inverse of the probability of participating in a single survey, given the individual’s personal characteristics. Non-response weights were re-scaled to the original study sample to avoid inflating the degrees-of-freedom. The final analysis weight is the product of all cross-sectional non-response weights and the single panel-attrition weight. Personal characteristics used in the weighting procedure included age, sex, occupation, resident status, length of stay in the village, and village. Additional follow-up characteristics used in the panel-attrition weight were a history of previous malaria, number of previous malaria episodes, previous positive rapid diagnostic test for malaria, and experience of adverse event in study. For both types of weights, we used the chi-square automatic interaction detection (*chaid*) approach to derive at groups of similar probability of participation [18]. We allowed the *chaid* procedure to expand the number of groups if there would be at least 25 observations in the resulting groups. Alpha-values for splitting and combining groups were set at 0.1. Inclusion of the predictors was unordered, while continuous variables were grouped in three similar-sized groups using tertiles. All statistical tests used an alpha level of 5%, below which statistical significance was assumed. All analyses used the original treatment assignment groups and were performed in STATA 14.1 (Stata Corp., College Station, Texas, USA).

### Ethics statement

The studies were approved by the Cambodian National Ethics Committee for Health Research (0029 NECHR, dated 04 Mar 2013), the Institute of Malariology, Parasitology and Entomology in Ho Chi Minh City (185/HDDD dated 15 May 2013), the Institute of Malariology, Parasitology and Entomology in Qui Nhon (dated 14 Oct 2013), the Lao National Ethics Committee for Health Research (Ref No 013-2015/NECHR), Government of the Lao PDR and the Oxford Tropical Research Ethics Committee (1015–13, dated 29 Apr 2013). Individual informed consent was obtained from each participant or parent/guardian in the case on minors. A fingerprint was obtained for illiterate participants countersigned by a literate witness. (ClinicalTrials.gov Identifier: NCT01872702)

## Results

Participant characteristics were similarly distributed in intervention and control villages (Table 1).

**Table 1:** Baseline sociodemographic, history of malaria and bednet use data of the control and intervention villages.

Characteristic	Control (deferred MDA) villages	Intervention (early MDA) villages	Overall
Cumulative number of participants	4,734	4,246	8,980
Number of first-time participation, n (%)			
Baseline	3,430 (72)	3,529 (83)	6,959 (77)
M03	558 (12)	316 (7)	874 (10)
M06	299 (6)	179 (4)	478 (5)
M09	238 (5)	130 (3)	368 (4)
M12	209 (4)	92 (2)	301 (3)
Age year, median (n, IQR)	20 (4,728, 8–35)	21 (4,241, 9–36)	20 (8,969, 9–36)
Sex, n (%)			
Male	2,412 (51)	2,196 (52)	4,608 (51)
Female	2,322 (49)	2,050 (48)	4,372 (49)
Occupation, n (%)			
Farmer	1,780 (38)	1,860 (44)	3,640 (41)
Child	413 (9)	266 (6)	679 (8)
Student	500 (11)	398 (9)	898 (10)
Others	194 (4)	225 (5)	419 (5)
Missing	1,847 (39)	1,497 (35)	3,344 (37)
History of malaria, n(%)			
Yes	754 (16)	758 (19)	1,512 (17)
No history of malaria	1,910 (40)	1,492 (35)	3,402 (38)
Bednet use, n(%)			
Regular	3,117 (66)	2,643 (62)	5,760 (64)
Irregular	470 (10)	555 (13)	1,025 (11)
Never use	90 (2)	122 (3)	212 (2)
Missing	1,057 (22)	926 (22)	1,983 (22)
Malaria infection by uPCR, n (%)			
*P*.*vivax*	432 (9)	281 (7)	713 (8)
*P*. *falciparum*	180 (4)	154 (4)	334 (4)
*P*. *vivax*+*P*. *falciparum*	94 (2)	57 (1)	151 (2)
Unidentified Plasmodium	137 (3)	165 (4)	302 (3)
Negative	3,531 (75)	3,188 (75)	6,719 (75)
Not done	360 (8)	401 (9)	761 (8)

MDA, mass drug administration; IQR, interquartile range; uPCR, ultrasensitive quantitative PCR.

### Coverage

**Fig 2:**
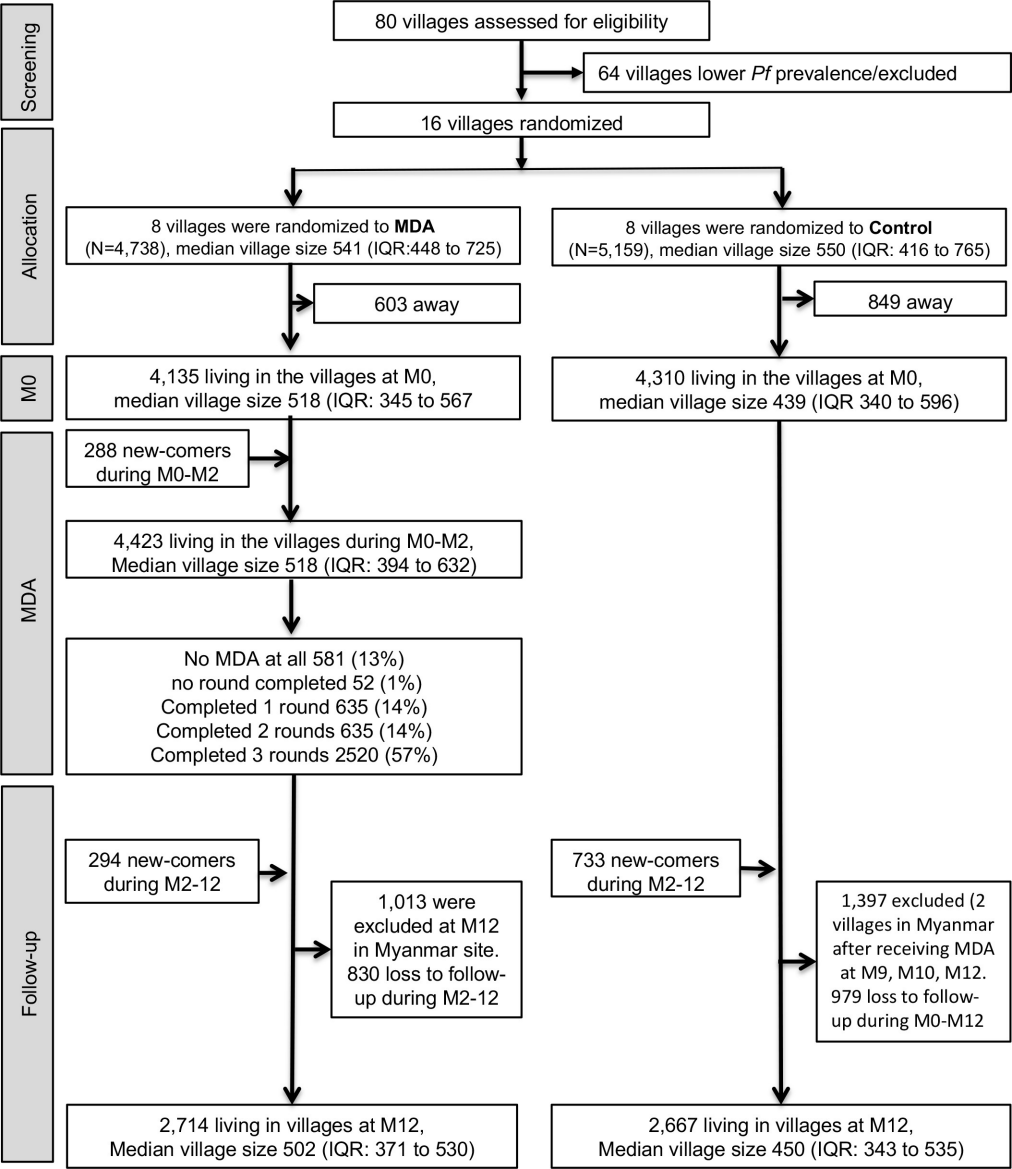
CONSORT flow diagram of MDA allocation and follow-up.

At baseline survey (M0), there were 4,135 and 4,310 people living in the intervention and control villages while 3,333/4,135 (80%) and 3,426/4,310 (79%) participated in the study, respectively. During three MDA rounds (M0, M1 and M2), 4,423 people were living in the intervention villages. Of those, 3,790 (86%) completed at least one round (3 doses) of DP-MDA and 2,520 (57%) completed 3 rounds (Fig 2).

### Effect on prevalence

**Fig 3:**
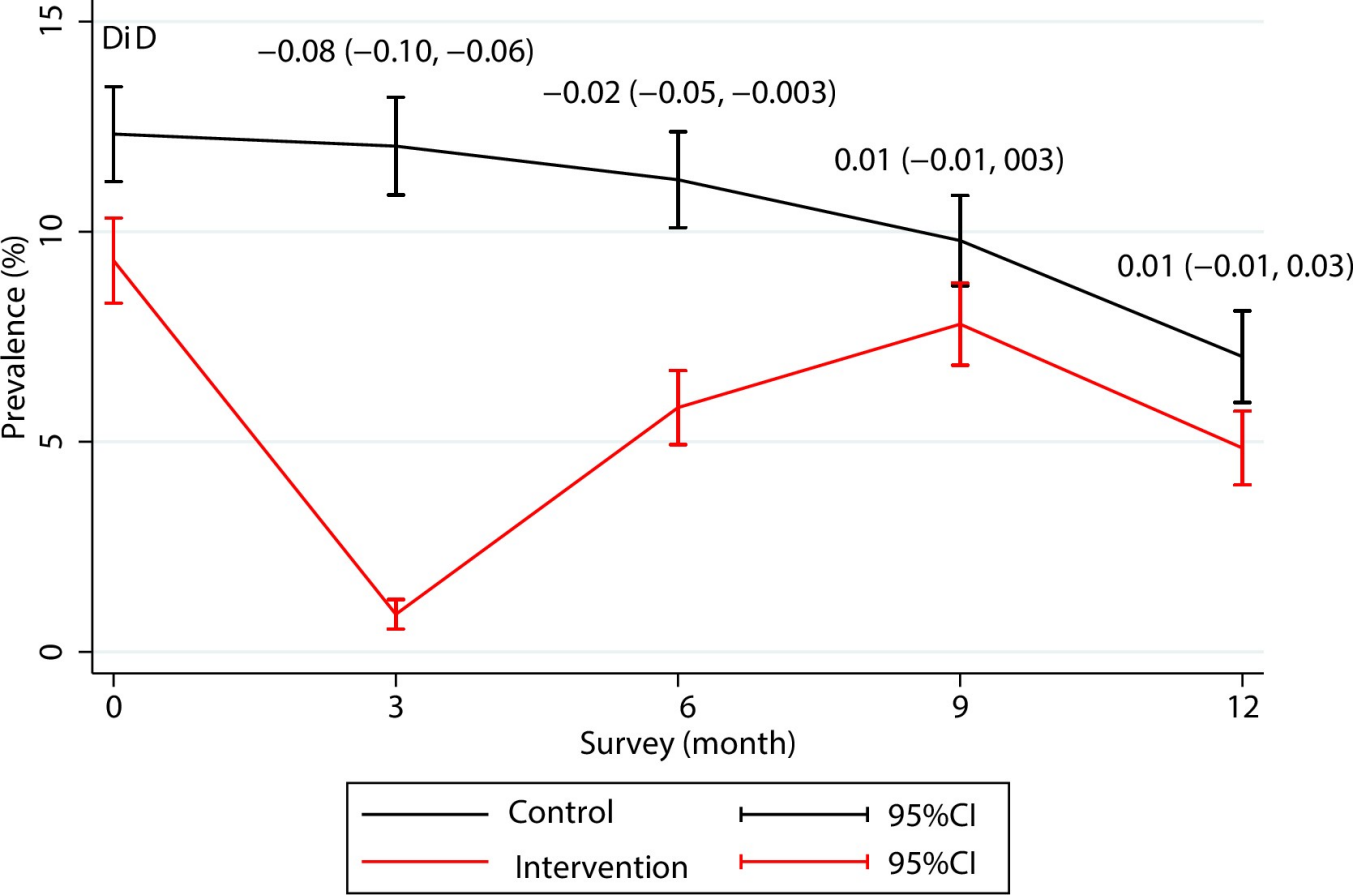
Changes in the prevalence of *P*. *vivax* infection during 12-month follow-up in the control and intervention villages. The month 12 data from Myanmar are not included because the cross-over MDA in the control villages had to be conducted on M9 instead of M12. DiD, difference in difference, coefficient (95% confident interval).

The prevalence of *P*. *vivax* parasitaemias decreased in both the intervention and control villages during the 12-month follow-up. The prevalence dropped sharply from 9.31% at baseline to 0.89% after three rounds of MDA at M3 follow-up (one month after the last MDA round) but rebounded to 5.81% by M6 follow-up after which the difference in prevalence between the intervention and the control villages was no longer statistically significant (coefficient (95%CI):-0.02 (-0.05, 0.003) (Fig 3). An analysis by country showed that the prevalence of *P*. *vivax* parasitaemias rebounded in each study site except for Lao PDR where the *P*. *vivax* prevalence dropped following the MDA and thereafter stayed low throughout the study period (S1 Fig).

### Effect on the incidence of *P*. *vivax* parasitaemias

**Fig 4:**
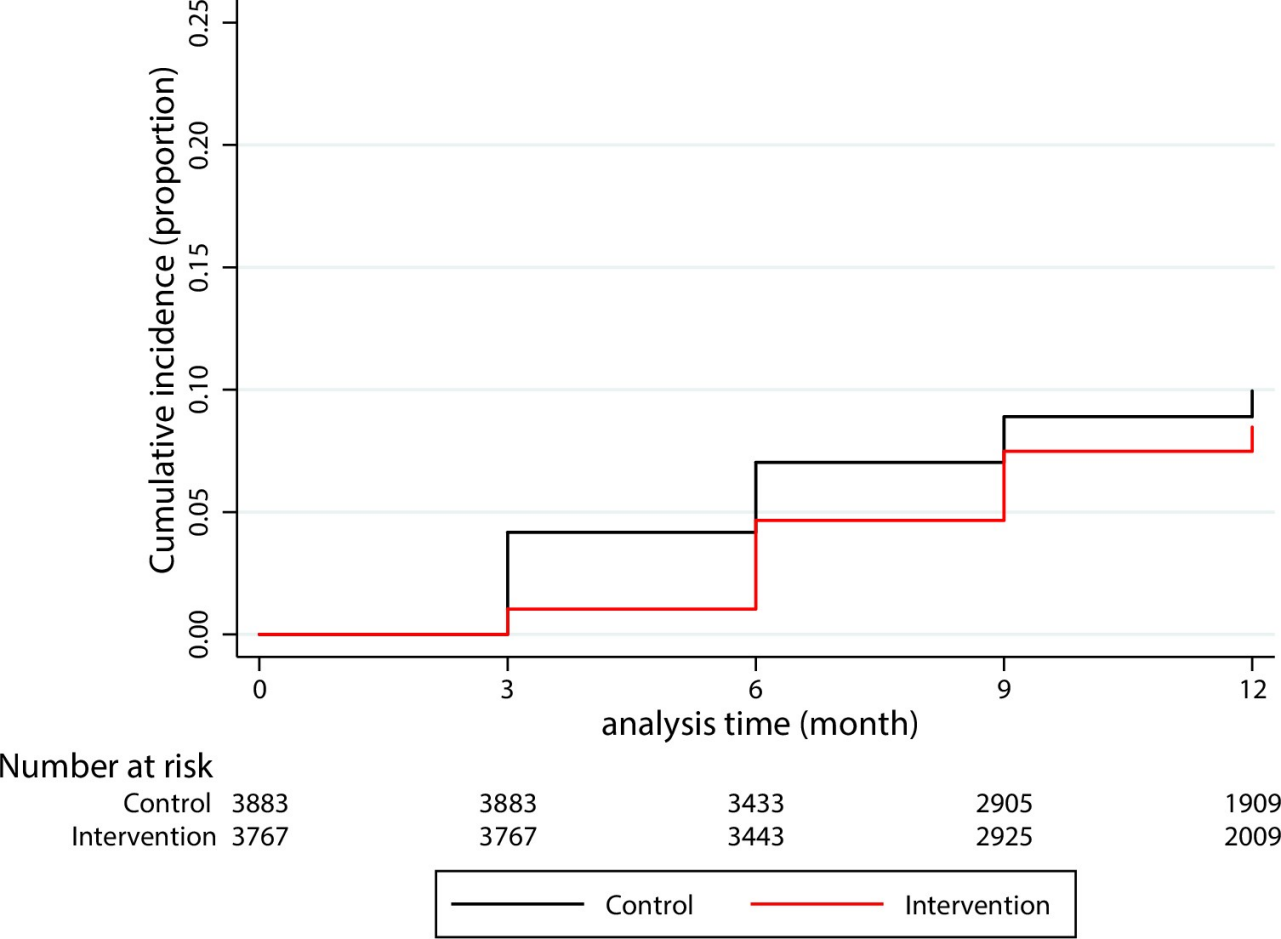
Cumulative uPCR-derived incidences of *P*. *vivax* infection between intervention and control villages. The month 12 data from Myanmar are not included because of logistic reasons, the cross-over MDA in the control villages had to be conducted on M9 instead of M12.

**Fig 5:**
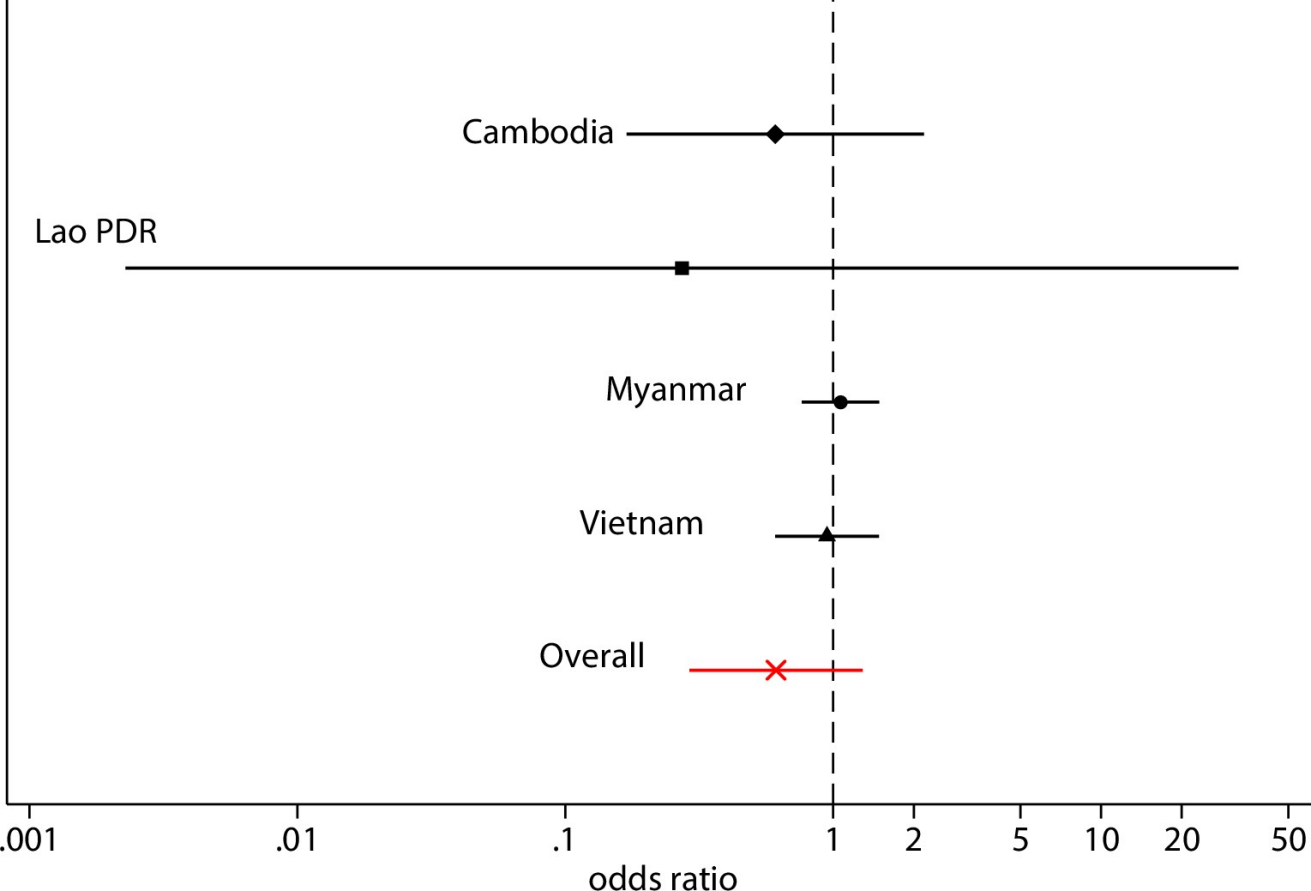
Forest plot of country odds ratios in the uPCR-derived incidence of *P*. *vivax* infections.

The Kaplan-Meier survival curves (Fig 4) show a slower and lower cumulative uPCR-derived incidence of *P*. *vivax* detected parasitaemias in the intervention group compared to the control group. The uPCR-derived incidence of *P*. *vivax* detected parasitaemias in the control villages by month 12 was highest in Vietnam, Lao PDR, and was lowest in Cambodia (S2 Fig). In Lao PDR the uPCR-derived *P*. *vivax* incidence of detected parasitaemias remained very low in the intervention villages over the follow-up period. In the other three sites, the incidence of detected parasitaemias in intervention villages rapidly approached the incidence seen in the control villages. There was no statistically significant evidence that the intervention reduced the cumulative incidence of *P*.*vivax* infection (95% confidence interval [CI] Odds ratio (OR): 0.29 to 1.36) within a year follow-up. The cumulative incidence of detectable *P*. *vivax* parasitaemias was 37% lower in the intervention group compared to the control group. This difference did not reach statistical significance and there is therefore no evidence of an effect of the intervention. This conclusion holds for each country separately (Fig 5). The intra-cluster correlation coefficient (ICC) for the incidence of detectable *P*. *vivax* parasitaemias or village (as a cluster), accounting for the random effect of country, was in the range from 0.17 to 0.63, estimated at baseline and every 3 months up to month 12. The weighted-average ICC was 0.55 over the one-year follow-up.

### Effect on recurrence of *P*. *vivax* parasitaemias and number of episodes detected

The number of participants with recurrent parasitaemias was lower in the intervention villages than in the control villages whether each positive uPCR tests was coded as a separate episode (OR: 0.34) or consecutive positive uPCR tests were coded together as one continuous episode (OR: 0.62). The estimates of the effect of MDA on recurrent parasitaemias varied-widely and did not reach statistical significance overall (95%CI: 0.08–1.42, and 0.18–2.12, respectively) (Table 2). Overall 809 (17.1%) of the participants in the control villages had at least one recurrent *P*. *vivax* episode compared to 562 (13.2%) in the intervention villages (Table 3). The sensitivity analyses defining missing value to negative or to positive showed similar outcomes (S2 & S3 Tables).

**Table 2:** Multilevel logistic regression with random effect for country and village on *P*. *vivax* episode and analysis weight. (panel attrition and non-response).

Variable	Control (deferred MDA) villages n = 4,734	Intervention (early MDA) villages n = 4,246	p-Value
**Each positive test = one episode, n**			
Participants with recurrence, n (%)	408 (8.6)	183 (4.3)	
Odds Ratio (95%CI)	Ref.	0.34 (0.08–1.42)	0.138
**Consecutive positive as one episode**			
Participants with recurrence, n (%)	170 (3.6)	113 (2.7)	
Odds Ratio (95%CI)	Ref.	0.62 (0.18–2.12)	0.443

MDA, mass drug administration; CI, confidence interval; Ref., reference

**Table 3:** Comparison of the number of *P*. *vivax* episodes in the control and intervention villages.

No. episodes	Each positive test = one episode		Consecutive positive test = one episode	
	Control n = 4,734	Intervention n = 4,246	Control n = 4,734	Intervention n = 4,246
	n (%)	n (%)	n (%)	n (%)
0	3925 (82.9)	3684 (86.8)	3925 (82.9)	3684 (86.8)
1	401 (8.5)	379 (8.9)	639 (13.5)	449 (10.6)
2	194 (4.1)	124 (2.9)	160 (3.4)	108 (2.5)
3	136 (2.9)	48 (1.1)	10 (0.2)	5 (0.1)
4	62 (1.3)	10 (0.2)		
5	16 (0.3)	1 (0)		

## Discussion

This study found no evidence of a sustained effect of MDA with dihydroartemisinin-piperaquine on the prevalence of *P*. *vivax* infections over a one-year follow-up. The MDA reduced the prevalence of *P*. *vivax* infections for three to six months after which a rebound in the prevalence of *P*. *vivax* parasitaemias was seen in all sites except for the sites in Lao PDR. The most likely reason for the absence of a rebound in Lao PDR is the lower *P*. *vivax* incidence compared to the other sites resulting in a lower risk for hypnozoite carriage, relapse and reinfection. Comparing *P*. *vivax* and *P*. *falciparum* prevalence changes between M3 and M6 suggests that the prevalence of *P*. *vivax* increased faster than the *P*. *falciparum* prevalence. One explanation for this observation could be that vivax hypnozoites fuel the speedy rebound of *P*. *vivax* prevalence. An alternative perhaps complementary explanation could be that the *P*. *vivax* transmission is higher at least in some of the study sites. MDA without a full course of 8-aminoquinolines clears only the blood stages of *P*. *vivax* and not the hypnozoites. As dihydroartemisinin-piperaquine clears *P*. *vivax* infections, recrudescent infections are unlikely the cause for recurrent *P*.*vivax* infections. Relapses, and to a lesser degree reinfections, are the most likely source for the observed *P*.*vivax* recurrences, and are a source of ongoing transmission [19]. In SE Asia, *P*. *vivax* infections tend to relapse after short intervals (typically three weeks), although the residual concentrations of slowly eliminated antimalarial drugs like piperaquine have a prophylactic effect which can lead to prolonged intervals [20]. Following the treatment of symptomatic infections with dihydroartemisinin-piperaquine, relapses emerge typically around six weeks after treatment [21, 22]. The radical cure of *P*. *vivax* infections requires a 7- or 14-day course of primaquine or single dose of tafenoquine. The SLDPQ aimed at blocking transmission by clearing *P*. *falciparum* gametocytes which in contrast to *P*. *vivax* gametocytes are resistant to schizontocidal drugs [23].

MDAs including radical cure with 8-aminoquinolines have been conducted in the former Soviet republics, Afghanistan and North Korea, and are thought to have contributed to the elimination of malaria from the USSR [24]. Mass primaquine administrations were used in Azerbaijan in 1971 to eliminate *P*. *vivax*. Despite the high prevalence of G6PD deficiency, primaquine administration was safe with few reported severe adverse events [25]. In China, primaquine mass administrations were implemented at a large scale in “spring treatment” involving over 200 million people at high risk for vivax malaria, and were effective in reducing the incidence of *P*. *vivax* malaria [26]. MDA with a three day-course of chloroquine and primaquine in Nicaragua showed a reduction of both *P*. *falciparum* and *P*. *vivax*, but *P*. *vivax* returned, as in our study, to endemic levels within months after the MDA [27].

Our study has limitations: 1) The intervention, MDA with DP and SLDPQ, was designed to eliminate *P*. *falciparum* and not *P*. *vivax* infections. Our post-hoc analyses describe the concurrent effect of the intervention targeting *P*. *falciparum* on *P*. *vivax*. 2) Treating villages in the uninterrupted presence of infective reservoirs in the surrounding areas will inevitably result in reimportation of infections, as we observed in the return of *P*. *falciparum* infections after 12 months [3]. A more regional approach to targeted malaria elimination including MDA would be required to reduce the risk of re-importation of Plasmodium infections. 3) The prevalence estimate at month 12 could be an underestimated due to the absence of data from Myanmar (where for logistic reasons the evaluation was conducted at month 9). In every site, except Laos, the prevalence of *P*. *vivax* increased between month 6 and 12 in the intervention villages [6]. 4) Entomological aspects were only evaluated in one study site [28]. Entomological data from more study sites may have helped to gain further insights into malaria transmission dynamics. 5) We sampled study participants in quarterly intervals. More frequent sampling would have provided a better understanding of the persistence of infections. The uPCR derived incidence of *P*. *vivax* parasitaemia reported in our study is much higher than the incidence of clinical vivax malaria but allows us to estimate the impact of the MDA on asymptomatic reservoir of *P*. *vivax* infections.

Our findings highlight the challenges but also suggest approaches to the elimination of all malarias including vivax malaria. The mass administration of dihydroartemisinin-piperaquine made a lasting and significant impact on *P*. *falciparum* infections [3], and on the *P*. *falciparum* entomological inoculation rate [28]. To provide a sustained reduction of *P*. *vivax* transmission, first and foremost radical cure with an 8-aminoquinoline is needed. Yet all 8-aminoquinolines cause haemolysis in G6PD deficient individuals. There are several approaches for the treatment of G6PD deficient individuals a) exclusion from the radical cure MDA b) use the one weekly primaquine 0.75mg/kg regimen for 8 weeks c) an alternative primaquine dosing regimen with incremental, yet safe increase in dosing. A regimen allowing primaquine to be stopped in case of symptoms or signs of haemolysis proved safe and effective without G6PD testing in the former USSR [25]. Pregnant women and young children should not receive 8-aminoquinolines. Robust and accurate point-of-care tests for the diagnosis of G6PD deficiency could play a pivotal role in the elimination of vivax malaria. A range of new diagnostic tests is currently in development [29, 30]. Second, high participation in mass treatment programmes is critical in clearing asymptomatic reservoirs. More than 80% of residents are thought having to participate in MDA to interrupt transmission but this estimate is not based on empirical data [31]. Although the coverage of at least one MDA round reached that target, the coverage of three MDA rounds was low. Finally, understanding the reservoirs of the nearby places and movement of people between places is essential in scaling up MDAs.

In conclusion, an MDA with schizontocidal and gametocytocidal medication for clearing only blood-stage infection made a short lasting impact on *P*. *vivax* prevalence and a reduction in recurrent infections but there no evidence of an effect on the long-term reduction of incidence, prevalence, and number of recurrent *P*. *vivax* infections over 12-month follow-up. This is in contrast to the impact of MDA on *P*. *falciparum* infections, where we showed a significantly longer lasting impact [3]. The combination of effective blood schizontocidal and hypnozoiticidal drugs will be essential for the elimination of all malarias.

## Supporting information

S1 CONSORT Checklist CONSORT 2010 checklist of information to include when reporting a randomised trial*. (DOC)

S1 Table The coding of number of recurrent episodes in 11 scenarios, green = uPCR negative and red = uPCR positive. (PDF)

S2 Table Multilevel logistic regression with random effect country and village on *P*. *vivax* recurrent episodes. (PDF)

S3 Table Comparison of the number of *P*. *vivax* episodes in control and intervention villages. (PDF)

S4 Table Number of *P*. *vivax* episodes in the control and intervention villages in Vietnam. (PDF)

S5 Table Number of *P*. *vivax* episodes in the control and intervention villages in Cambodia. (PDF)

S6 Table Number of *P*. *vivax* episodes in the control and intervention villages in Lao PDR. (PDF)

S7 Table Number of *P*. *vivax* episodes in the control and intervention villages in Myanmar. (PDF)

S1 Fig *P*. *vivax* prevalence. (PDF)

S2 Fig (PDF)

S1 Protocol (DOCX)
