# Supplementary material for: Mass drug administrations with dihydroartemisinin-piperaquine and single low dose primaquine to eliminate Plasmodium falciparum have only a transient impact on Plasmodium vivax: Findings from randomised controlled trials
Source: PLoS One. 2020 Feb 5;15(2):e0228190. doi: 10.1371/journal.pone.0228190 (PMC7001954; doi:10.1371/journal.pone.0228190)
Supplement: S1 Table — (PDF) [file pone.0228190.s002.pdf]

Table S1: The coding of number of recurrent episodes in 11 scenarios, Green=uPCR negative and Red=uPCR positive

Scenario	PCR test result per survey round 1 to 5: green = negative, red = positive					Interpretation: Counts of positive episodes	
	1	2	3	4	5	If each test is considered as a single episode	If consecutive positives are considered single episodes
1						0	0
2						1	1
3						2	2
4						2	1
5						3	3
6						3	2
7						3	1
8						4	2
9						4	2
10						4	1
11						5	1
