# Supplementary material for: Mass drug administrations with dihydroartemisinin-piperaquine and single low dose primaquine to eliminate Plasmodium falciparum have only a transient impact on Plasmodium vivax: Findings from randomised controlled trials
Source: PLoS One. 2020 Feb 5;15(2):e0228190. doi: 10.1371/journal.pone.0228190 (PMC7001954; doi:10.1371/journal.pone.0228190)
Supplement: S2 Table — (PDF) [file pone.0228190.s003.pdf]

Table S2: Multilevel logistic regression with random effect country and village on *P. vivax* recurrent episodes

Variable	Available data		Missing test = positive		Missing test = negative	
	Control	Intervention	Control	Intervention	Control	Intervention
**Each positive test = one episode**	N = 4,734	N = 4,246	N = 4,734	N = 4,246	N = 4,734	N = 4,246
Participants with recurrence, n(%)	408 (8.62)	183 (4.31)	575 (12.15)	366 (8.62)	408 (8.62)	183 (4.31)
OR (95%CI)	1	0.34 (0.08-1.42)	1	0.46 (0.13-1.62)	1	0.34 (0.08-1.42)
p-Value		0.138		0.227		0.138
**Consecutive positive as one episode**						
Participants with recurrence, n(%)	170 (3.59)	113 (2.66)	263 (5.56)	209 (4.92)	170 (3.59)	113 (2.66)
OR (95%CI)	1	0.62 (0.18-2.12)	1	0.67 (0.22-2.03)	1	0.62 (0.18-2.12)
p-Value		0.443		0.475		0.443

Control, deferred-MDA villages; Intervention, early-MDA villages; CI, confidence interval
