# Supplementary material for: Mass drug administrations with dihydroartemisinin-piperaquine and single low dose primaquine to eliminate Plasmodium falciparum have only a transient impact on Plasmodium vivax: Findings from randomised controlled trials
Source: PLoS One. 2020 Feb 5;15(2):e0228190. doi: 10.1371/journal.pone.0228190 (PMC7001954; doi:10.1371/journal.pone.0228190)
Supplement: S3 Table — (PDF) [file pone.0228190.s004.pdf]

Table S3: Comparison of the number of *P. vivax* episodes in control and intervention villages

	Each positive test = one episode											
	Available data				Missing test = positive				Missing test = negative			
	Control N=4734		Intervention N=4246		Control N=4734		Intervention N=4246		Control N=4734		Intervention N=4246	
**Number episodes**	n	%	n	%	n	%	n	%	n	%	n	%
0	3925	82.9	3684	86.8	3240	68.4	3126	73.6	3925	82.9	3684	86.8
1	401	8.5	379	8.9	919	19.4	754	17.8	401	8.5	379	8.9
2	194	4.1	124	2.9	311	6.6	243	5.7	194	4.1	124	2.9
3	136	2.9	48	1.1	174	3.7	103	2.4	136	2.9	48	1.1
4	62	1.3	10	0.2	72	1.5	18	0.4	62	1.3	10	0.2
5	16	0.3	1	0	18	0.4	2	0	16	0.3	1	0
	**Consecutive positive test = one episode**											
	**Available data**				**Missing test = positive**				**Missing test = negative**			
	Control N=4734		Intervention N=4246		Control N=4734		Intervention N=4246		Control N=4734		Intervention N=4246	
**Number episodes**	n	%	n	%	n	%	n	%	n	%	n	%
0	3925	82.9	3684	86.8	3240	68.4	3126	73.6	3925	82.9	3684	86.8
1	639	13.5	449	10.6	1231	26	911	21.5	639	13.5	449	10.6
2	160	3.4	108	2.5	250	5.3	201	4.7	160	3.4	108	2.5
3	10	0.2	5	0.1	13	0.3	8	0.2	10	0.2	5	0.1
