# Supplementary material for: Mass drug administrations with dihydroartemisinin-piperaquine and single low dose primaquine to eliminate Plasmodium falciparum have only a transient impact on Plasmodium vivax: Findings from randomised controlled trials
Source: PLoS One. 2020 Feb 5;15(2):e0228190. doi: 10.1371/journal.pone.0228190 (PMC7001954; doi:10.1371/journal.pone.0228190)
Supplement: S4 Table — (PDF) [file pone.0228190.s005.pdf]

Table S4: Number of *P. vivax* episodes in the control and intervention villages in Vietnam

**Vietnam**	**Each positive test = one episode**											
	**Available data**				**Missing = positive**				**Missing = negative**			
**Episodes**	Control N = 1411		Intervention N = 1525		Control N = 1411		Intervention N = 1525		Control N = 1411		Intervention N = 1525	
	n	%	n	%	n	%	n	%	n	%	n	%
0	1206	85.5	1315	86.2	823	58.3	1036	67.9	1206	85.5	1315	86.2
1	105	7.4	124	8.1	431	30.5	347	22.8	105	7.4	124	8.1
2	43	3	48	3.1	89	6.3	86	5.6	43	3	48	3.1
3	33	2.3	28	1.8	38	2.7	41	2.7	33	2.3	28	1.8
4	18	1.3	9	0.6	22	1.6	13	0.9	18	1.3	9	0.6
5	6	0.4	1	0.1	8	0.6	2	0.1	6	0.4	1	0.1
	**Consecutive tests = one episode**											
	**Available data**				**Missing = positive**				**Missing = negative**			
**Episodes**	Control N = 1411		Intervention N = 1525		Control N = 1411		Intervention N = 1525		Control N = 1411		Intervention N = 1525	
	n	%	n	%	n	%	n	%	n	%	n	%
0	1206	85.5	1315	86.2	823	58.3	1036	67.9	1206	85.5	1315	86.2
1	163	11.6	164	10.8	489	34.7	399	26.2	163	11.6	164	10.8
2	38	2.7	43	2.8	92	6.5	84	5.5	38	2.7	43	2.8
3	4	0.3	3	0.2	7	0.5	6	0.4	4	0.3	3	0.2
