# Supplementary material for: Mass drug administrations with dihydroartemisinin-piperaquine and single low dose primaquine to eliminate Plasmodium falciparum have only a transient impact on Plasmodium vivax: Findings from randomised controlled trials
Source: PLoS One. 2020 Feb 5;15(2):e0228190. doi: 10.1371/journal.pone.0228190 (PMC7001954; doi:10.1371/journal.pone.0228190)
Supplement: S5 Table — (PDF) [file pone.0228190.s006.pdf]

Table S5: Number of *P. vivax* episodes in the control and intervention villages in Cambodia

	Each positive test = one episode											
**Cambodia**	**Available data**				**Missing = positive**				**Missing = negative**			
**Episodes**	Control N = 1097		Intervention N = 757		Control N = 1097		Intervention N = 757		Control N = 1097		Intervention N = 757	
	n	%	n	%	n	%	n	%	n	%	n	%
0	972	88.6	676	89.3	873	79.6	621	82	972	88.6	676	89.3
1	47	4.3	51	6.7	124	11.3	85	11.2	47	4.3	51	6.7
2	29	2.6	14	1.8	41	3.7	25	3.3	29	2.6	14	1.8
3	32	2.9	15	2	38	3.5	24	3.2	32	2.9	15	2
4	13	1.2	1	0.1	17	1.5	2	0.3	13	1.2	1	0.1
5	4	0.4	0	0	4	0.4	0	0	4	0.4	0	0
	**Consecutive positive tests = one episode**											
	**Available data**				**Missing = positive**				**Missing = negative**			
**Episodes**	Control N = 1097		Intervention N = 757		Control N = 1097		Intervention N = 757		Control N = 1097		Intervention N = 757	
	n	%	n	%	n	%	n	%	n	%	n	%
0	972	88.6	676	89.3	873	79.6	621	82	972	88.6	676	89.3
1	87	7.9	57	7.5	170	15.5	106	14	87	7.9	57	7.5
2	36	3.3	22	2.9	52	4.7	28	3.7	36	3.3	22	2.9
3	2	0.2	2	0.3	2	0.2	2	0.3	2	0.2	2	0.3
