# Supplementary material for: Mass drug administrations with dihydroartemisinin-piperaquine and single low dose primaquine to eliminate Plasmodium falciparum have only a transient impact on Plasmodium vivax: Findings from randomised controlled trials
Source: PLoS One. 2020 Feb 5;15(2):e0228190. doi: 10.1371/journal.pone.0228190 (PMC7001954; doi:10.1371/journal.pone.0228190)
Supplement: S6 Table — (PDF) [file pone.0228190.s007.pdf]

Table S6: Number of *P. vivax* episodes in the control and intervention villages in Lao PDR

**Laos**	**Each positive test = one episode**											
	**Available data**				**Missing = positive**				**Missing = negative**			
**Episodes**	Control N = 877		Intervention N = 949		Control N = 877		Intervention N = 949		Control N = 877		Intervention N = 949	
	n	%	n	%	n	%	n	%	n	%	n	%
0	712	81.2	924	97.4	689	78.6	905	95.4	712	81.2	924	97.4
1	71	8.1	24	2.5	87	9.9	40	4.2	71	8.1	24	2.5
2	43	4.9	1	0.1	49	5.6	4	0.4	43	4.9	1	0.1
3	29	3.3	0	0	30	3.4	0	0	29	3.3	0	0
4	16	1.8	0	0	16	1.8	0	0	16	1.8	0	0
5	6	0.7	0	0	6	0.7	0	0	6	0.7	0	0
	**Consecutive tests = one episode**											
	**Available data**				**Missing = positive**				**Missing = negative**			
**Episodes**	Control N = 877		Intervention N = 949		Control N = 877		Intervention N = 949		Control N = 877		Intervention N = 949	
	n	%	n	%	n	%	n	%	n	%	n	%
0	712	81.2	924	97.4	689	78.6	905	95.4	712	81.2	924	97.4
1	127	14.5	24	2.5	146	16.6	41	4.3	127	14.5	24	2.5
2	34	3.9	1	0.1	38	4.3	3	0.3	34	3.9	1	0.1
3	4	0.5	0	0	4	0.5	0	0	4	0.5	0	0
