# Supplementary material for: Mass drug administrations with dihydroartemisinin-piperaquine and single low dose primaquine to eliminate Plasmodium falciparum have only a transient impact on Plasmodium vivax: Findings from randomised controlled trials
Source: PLoS One. 2020 Feb 5;15(2):e0228190. doi: 10.1371/journal.pone.0228190 (PMC7001954; doi:10.1371/journal.pone.0228190)
Supplement: S7 Table — (PDF) [file pone.0228190.s008.pdf]

Table S7: Number of P. vivax episodes in the control and intervention villages in Myanmar

Myanmar	Each positive test = one episode											
	Available data				Missing = positive				Missing = negative			
Episodes	Control N = 1349		Intervention N =1015		Control N = 1349		Intervention N =1015		Control N = 1349		Intervention N =1015	
	n	%	n	%	n	%	n	%	n	%	n	%
0	1035	76.7	769	75.8	855	63.4	564	55.6	1035	76.7	769	75.8
1	178	13.2	180	17.7	277	20.5	282	27.8	178	13.2	180	17.7
2	79	5.9	61	6	132	9.8	128	12.6	79	5.9	61	6
3	42	3.1	5	0.5	68	5	38	3.7	42	3.1	5	0.5
4	15	1.1	0	0	17	1.3	3	0.3	15	1.1	0	0
	Consecutive tests = one episode											
	Available data				Missing = positive				Missing = negative			
Episodes	Control N = 1349		Intervention N =1015		Control N = 1349		Intervention N =1015		Control N = 1349		Intervention N =1015	
	n	%	n	%	n	%	n	%	n	%	n	%
0	1035	76.7	769	75.8	855	63.4	564	55.6	1035	76.7	769	75.8
1	262	19.4	204	20.1	426	31.6	365	36	262	19.4	204	20.1
2	52	3.9	42	4.1	68	5	86	8.5	52	3.9	42	4.1
