# Supplementary material for: Mass drug administrations with dihydroartemisinin-piperaquine and single low dose primaquine to eliminate Plasmodium falciparum have only a transient impact on Plasmodium vivax: Findings from randomised controlled trials
Source: PLoS One. 2020 Feb 5;15(2):e0228190. doi: 10.1371/journal.pone.0228190 (PMC7001954; doi:10.1371/journal.pone.0228190)

Figure S2: *P. vivax* incidence Cambodia

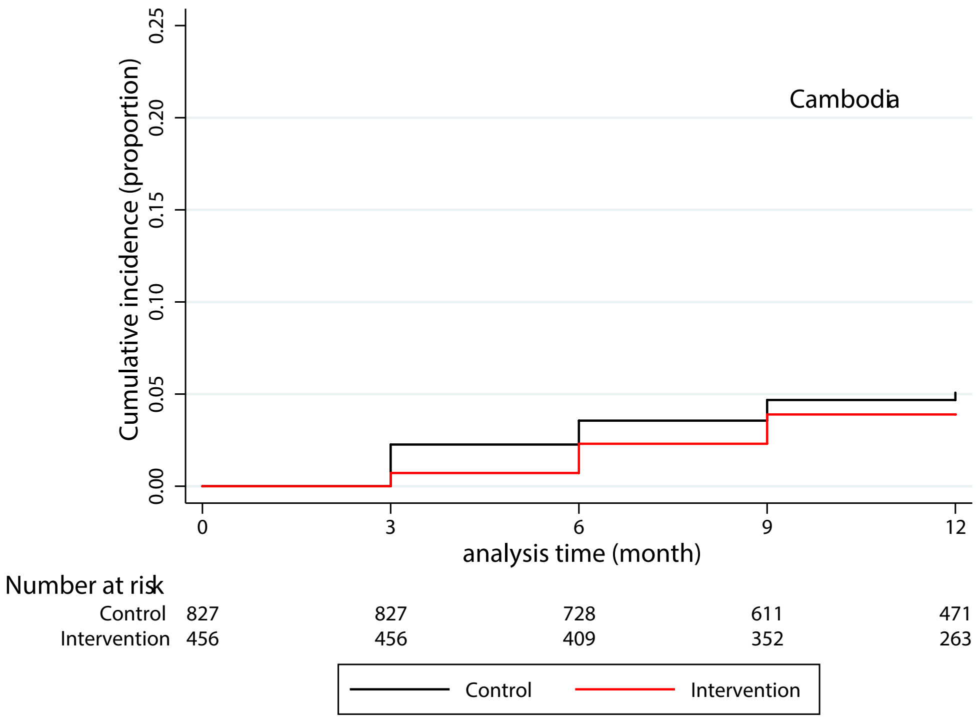

Figure S2: ***P. vivax*** **incidence Lao PDR**

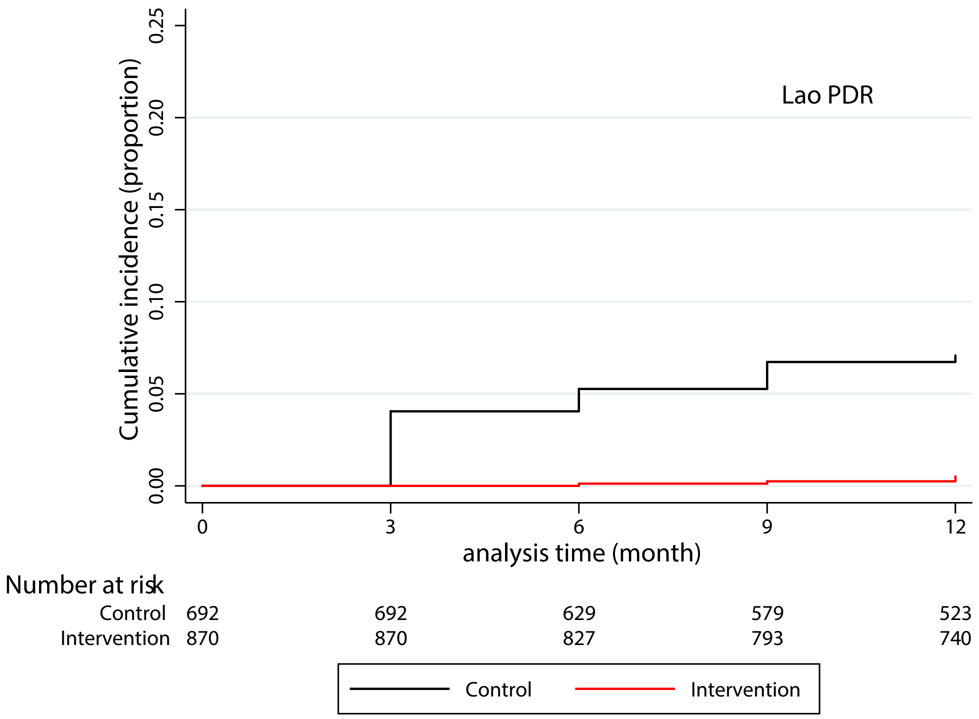

Figure S2: *P. vivax* incidence Myanmar

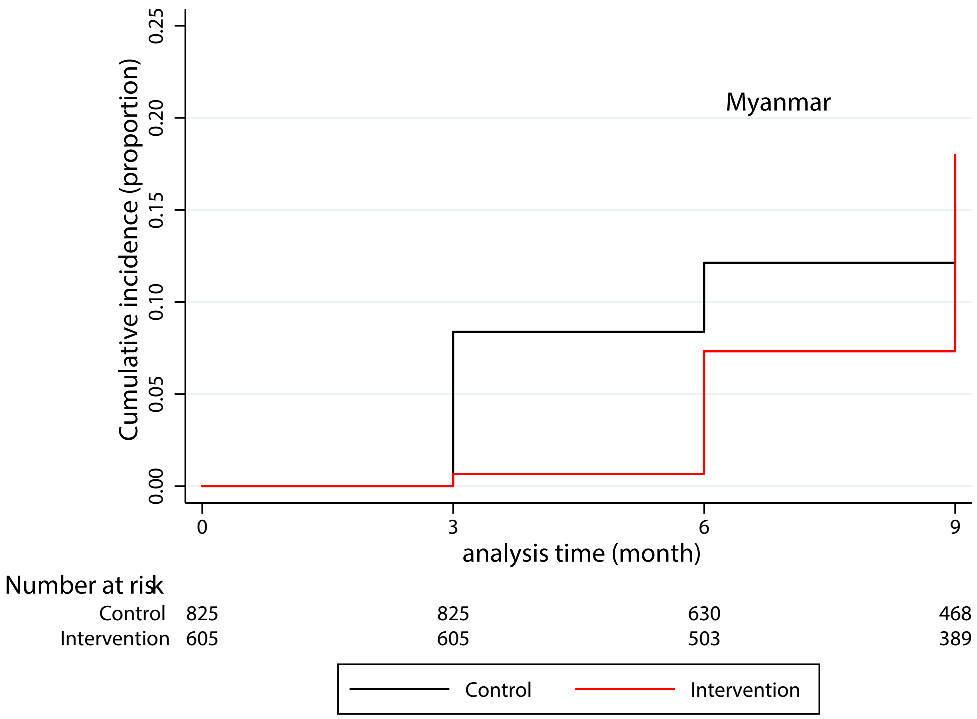

Figure S2: *P. vivax* incidence Vietnam

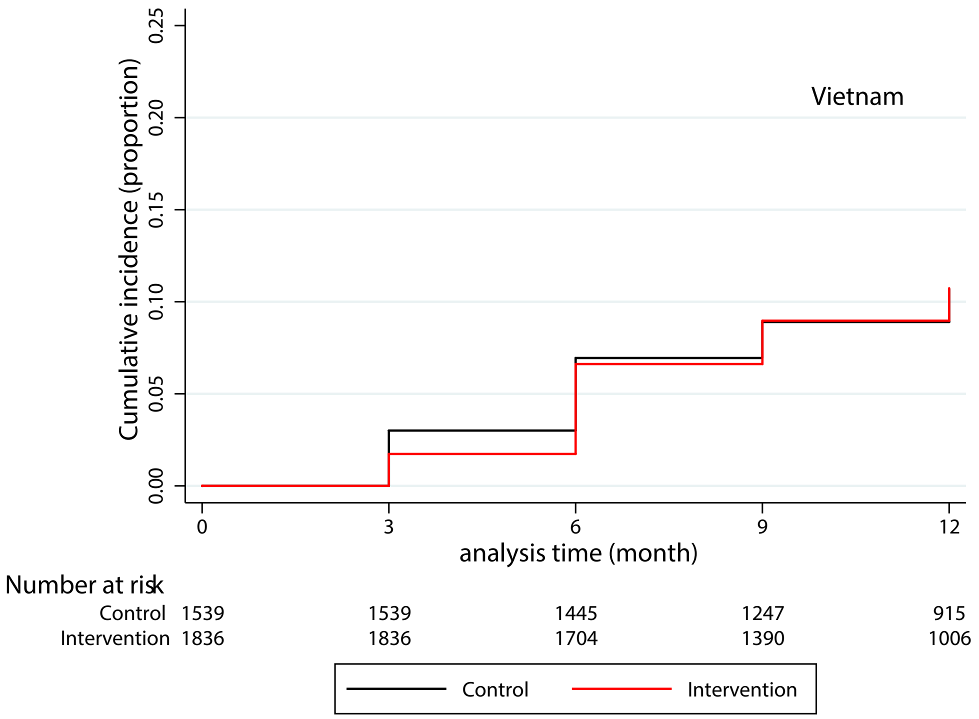

Supplement: S2 Fig — (PDF) [file pone.0228190.s010.pdf]
