# Supplementary material for: Mass drug administrations with dihydroartemisinin-piperaquine and single low dose primaquine to eliminate Plasmodium falciparum have only a transient impact on Plasmodium vivax: Findings from randomised controlled trials
Source: PLoS One. 2020 Feb 5;15(2):e0228190. doi: 10.1371/journal.pone.0228190 (PMC7001954; doi:10.1371/journal.pone.0228190)
Supplement: S1 Protocol — (DOCX) [file pone.0228190.s011.docx]

**Targeted chemo-elimination (TCE) to eradicate malaria in areas of suspected or proven artemisinin resistance in Southeast Asia and South Asia**

**Principal Investigator:** Professor Nicholas J White

**Sponsor:** University of Oxford

**Funder:** Bill and Melinda Gates Foundation (BMGF) & the Wellcome Trust of Great Britain

**Sites and corresponding site principal investigators**

**1) Thai-Myanmar border:** Myawadi township, Myanmar; Professor François Nosten, Shoklo Malaria Research Unit

**2) Vietnam:** Binh Phuoc Province; Professor Tran Tinh Hien, Oxford University Clinical Research Unit, Viet Nam

**3) Cambodia**: Pailin;, Dr. C. Nguon, National Malaria Centre, Cambodia; Prof. A. Dondorp, Dr. R. Tripura, Dr T. Peto MORU, Thailand.

**4) Myanmar:** Sites to be confirmed; Dr. Kyaw Myo Tun, MOCRU, Myanmar.

5) Bangladesh: Sites to be confirmed; Professor Abul Faiz, Malaria Research Group, Bangladesh

6) **Laos:** Savanakhet Province; Dr Mayfong Mayxay and Prof. Paul Newton, LOMWRU, Mahosot Hospital, Laos

And Mahidol Oxford Tropical Medicine Research Unit (MORU): Professor Nicholas Day, Dr Lorenz von Seidlein, Professor Arjen Dondorp

**Confidentiality Statement**

This document contains confidential information that must not be disclosed to anyone other than the sponsor, the investigator team, host institution, relevant ethics committee and regulatory authorities.

**TABLE OF CONTENTS**

LIST OF ABBREVIATIONS 3

I. BACKGROUND 4

II. OBJECTIVES 5

III. PRIMARY AND SECONDARY ENDPOINTS 5

1. Primary endpoint 5

2. Secondary endpoints 5

IV. STUDY DESIGN 6

1. Type of study 6

2. Study sites and duration 6

3. Study populations, sample size and statistical considerations 7

V. PROCEDURES 7

1. Procedures timeline 7

1.1 Pre-TCE activities including community engagement 8

1.2 First exhaustive cross-sectional surveys and first TCE round 8

1.3 Second and third rounds of TCE and cross-sectional surveys 9

1.4 Post-TCE survey 9

1.5 Follow up 9

1.6 Second year 10

2. Type and timing of surveys 10

2.1 Cross sectional surveys 10

2.2 Other surveys 10

2.3 Sample processing 10

3. Drugs 11

4. TCE protocol 11

4.1 Inclusion criteria 12

4.2 Exclusion criteria 12

4.3 Withdrawal criteria 12

4.4 Informed consent and eligibility 12

5. Evaluation of participants’ perception, tolerability, safety and acceptability of TCE 12

VI. QUALITY CONTROL AND ASSURANCE PROCEDURES 13

VII. ETHICS 13

VIII. DATA HANDLING, ACCESS AND PUBLICATIONS 14

IX. REFERENCES 15

# LIST OF ABBREVIATIONS

ACT Artemisinin-based combination therapy

AE Adverse event

CRF Case record form

DP Dihydroartemisinin-piperaquine

FGD Focus group discussion

G6PD Glucose-6-phosphate dehydrogenase

GCP Good Clinical Practice

GIS Geographic information system

GPS Global positioning system

Hb Haemoglobin

IDI In-depth interview

MDA Mass drug administration

MORU Mahidol-Oxford Tropical Medicine Research Unit

MPAC Malaria Policy Advisory Committee

NMCP National Malaria Control Programme

PCR Polymerase Chain Reaction

PQ Primaquine

RDT Rapid diagnostic test

SAE Serious adverse event

SEA South East Asia

SMRU Shoklo Malaria Research Unit

SOP Standard Operating Procedure

TCE Targeted chemo-elimination

WHO World Health Organisation

WWARN Worldwide Antimalarial Resistance Network

# I. BACKGROUND

The spread of artemisinin resistance in *Plasmodium falciparum,* which compromises the therapeutic efficacy of artemisinin combination treatments (ACTs), is the greatest threat to current global initiatives to control and eliminate malaria and is considered the highest priority of the WHO Global Malaria Programme [1]. If not eliminated, resistant parasites could spread across Asia to Africa, as happened with resistance to other antimalarials in the past. Conventional descriptions of the epidemiology of malaria in low transmission settings suggest that malaria prevalences are low (<10%) and heterogeneous. Most or all infections are thought to be symptomatic so the focus of malaria control activities is on the identification and treatment of symptomatic individuals. We and others have shown recently that artemisinin resistant *P. falciparum* is prevalent in Western Cambodia, and that it is now also found along the Thailand-Myanmar border and Vietnam [2,3,4,5]. We have recently developed highly sensitive quantitative PCR (qPCR) methods for parasite detection using >1mL of blood which are 5,000 times more sensitive than conventional microscopy, and 100 times more sensitive than currently used PCR. We have studied villages along the Thai-Myanmar border which are typical for the region and are classified by conventional epidemiological techniques as low-transmission (5-20% malaria prevalence). Our studies suggest that the majority of the population is infected. In Pailin, Western Cambodia, in areas where the National Malaria Control Programme and WHO believe that malaria has been all but eliminated, we have also found very high rates (>80%) of sub-microscopic parasitaemia in patients with fever or history of fever who are RDT negative. Thus, there is a lot more asymptomatic malaria in low transmission settings than previously thought, suggesting that control and elimination activities need to be rethought.

This study is designed to conduct and evaluate the efficacy of pilot implementation of targeted chemo-elimination (TCE) in selected areas with the goal of eliminating malaria in these regions. TCE differs from mass drug administration (MDA); it is a strategy used to identify specific areas where mass treatment is necessary, in this case to eliminate all malaria parasites. TCE will be targeted at communities with significant levels of subclinical infection and transmission which will be identifiable in the future by comparing rates of positivity by RDT or microscopy from new population samples against our qPCR data, which shows the true falciparum prevalence. The study will assess the feasibility, safety and acceptability of TCE and its impact on the transmission of malaria and the progression of artemisinin resistance. In addition it will evaluate the contribution of low parasitaemia carriage to transmission of artemisinin resistant malaria. These pilot studies are a necessary prelude to future scale up and policy implementation.

Dihydroartemisinin-piperaquine (DP) is a highly efficacious and inexpensive ACT which is well tolerated by all age groups when used to treat uncomplicated multidrug resistant falciparum malaria in South East Asia [6,7,8,9]. Monthly DP treatments have proved highly effective and well tolerated [3]. When used as part of a MDA strategy, the addition of a gametocytocidal drug contributes towards the goal of malaria elimination by adding a strong transmission blocking activity to the regimen. Primaquine (PQ), the only currently licensed 8-aminoquinoline, is relatively safe and very effective when used at a dose of 0.25 mg base/kg, and does not require G6PD screening [10]. Thus, we propose to evaluate the potential of a TCE programme with to eliminating malaria focally in areas where artemisinin resistance in *P. falciparum* is prevalent using DP plus PQ.

# II. OBJECTIVES

The overall aim of this study is to pilot-implement TCE in known areas of artemisinin resistance to evaluate its potential in eliminating falciparum malaria and therefore artemisinin resistance. Specifically the study will focus on the following objectives:

**Primary objective:**

To assess the feasibility, safety, acceptability, efficacy and effectiveness of TCE with DP + PQ as a tool for eliminating falciparum malaria

**Secondary objectives:**

1. Assess the efficacy of TCE in eliminating the malaria parasite reservoir
2. Assess the impact of TCE on malaria transmission
3. Describe the feasibility, safety and acceptability of TCE
4. Characterize parasite carriage using highly sensitive techniques in four geographically separate sites where resistance to artemisinin has been documented.
5. Assess the contribution of sub-microscopic parasite carriage to the transmission of artemisinin resistant parasite strains

# III. PRIMARY AND SECONDARY ENDPOINTS

## Primary endpoint

The primary endpoint will be the prevalence of falciparum malaria measured by qPCR, 12 months after the first administration of TCE.

## Secondary endpoints

Secondary endpoints will be:

- Safety and acceptability of TCE evaluated by questionnaires filled out by participants or care givers
- Effect on gametocyte carriage by TCE (measured by the proportions of gametocyte carriers over the 12 month period)
- The effect of TCE on the incidence of clinical malaria in the villages over the first 12 months
- Molecular analysis of parasite genotypes, markers of resistance and parasite population genetic structure
- Acceptability of TCE in the four study villages
- Cost estimates of TCE by sampling strategy

# IV. STUDY DESIGN

## Type of study

This is a cluster randomized, controlled, not-blinded clinical trial including residents of four Lao villages. Two villages will receive the intervention (TCE) and the other two will be the controls. The control villages will help explain non-TCE related trends in malaria prevalence over the year such as seasonal effects as well as changes in detectable falciparum over the year in the absence of treatment and on the infectivity of non-clinical parasitaemia. Five or more villages will be pre-surveyed first leading to selection of four villages per site, of which two will be randomised to TCE and two to non-TCE control. The control villages will be TCE treated the following year should TCE prove successful.

## Study sites and duration

TCE will be pilot-implemented in four sites and its effects and falciparum malaria-elimination potential will be evaluated over the course of one year; there will be two TCE villages per site and two non-TCE villages which will serve as controls. The study has been implemented in two sites on the Thai-Myanmar border and Vietnam during the first year of the project . Sites in Myanmar, Laos and Bangladesh will be added in 2015.

Prior to the beginning of the pilot-implementation of TCE in the first year, all sites will be pre-surveyed for selection of the TCE and control villages: five or more candidate villages in each region will be visited prior to TCE pilot-implementation and 50 random 2 mL blood samples per village will be taken from adults to estimate the prevalence of submicroscopic infections; 4 villages will be chosen based on a dry season malaria prevalence >10% for piloting TCE implementation during the first year. All malarias will be includes independent of the Plasmodium species. In very low density infections it is often not possible to determine the species. Once chosen, the villages will be visited regularly to engage with and prepare the community. This work leading up to the main TCE implementation will be documented as it is essential to ensure that TCE coverage will be as close as 100% as possible after excluding individuals based on appropriate exclusion criteria.

## Study populations, sample size and statistical considerations

Four villages in total per site will be identified in regions where artemisinin resistance has been documented. Each village will be of about 250-1200 people each.. Villages will be selected based on quantitative reasons (prevalence parasitaemia) and qualitative reasons (expected enthusiasm of the residents resulting in an adequate coverage). Two out of the four villages in each site will be randomized to receive TCE, the remaining two will receive no treatment (control villages).

The primary objective of this study is to test the feasibility and acceptability of implementing TCE in the six study areas, as well as its safety, efficacy and effectiveness in reducing asymptomatic malaria prevalence. Hence the primary analysis will be of the percentage falls in asymptomatic malaria prevalence in the TCE villages as determined by highly sensitive RT-PCR. We hope to see 95% reductions in prevalence rates in all sites at the end of one year.

To ensure that any reductions seen are not due to changes in endemicity unrelated to the TCE, we will also conduct a formal overall analysis comparing changes in prevalence in each study area between the TCE villages and non-TCE control villages. There will be a total of at least 4 villages in each area. For 80% power to detect a 95% fall in prevalence from a 10% initial prevalence (controlled for random changes in prevalence in the control groups) there will be a minimum required participants to be recruited and followed up of at least 152 individuals in each village. Analysis will be as a cluster randomised, repeated measures RCT, using a hierarchical logistical regression model accounting for between area (country), between cluster and between individual effects.

# V. PROCEDURES

## Procedures timeline

### 1.1 Pre-TCE activities including community engagement

Potential villages will be visited and meetings organized with the local population and their leaders to explain the objectives of the project and to understand whether participation is acceptable and feasible. Community consent will be needed to proceed further. Five or more candidate villages, each with stable population sizes of about 250-1200 in each site will be selected based on criteria such as: easy access for community engagement and located where samples can reach the laboratories in less than 24 hours before processing. Once the study villages are chosen they will be visited regularly to engage with and prepare the community. A census will be performed and houses, work areas, and relevant migration routes will be GIS mapped. The final four villages per site will be chosen for TCE implementation based on a dry season malaria prevalence > 10%. Two of the four villages will be non-TCE control villages, two TCE. The control villages will be informed that they will receive TCE in the second year if the pilot TCE proves successful. After consultations and community engagement, health posts in each village will be set up. A presurvey where 1.5ml blood samples from 50 healthy adult volunteers in up to 20 villages will be conducted to establish sub-clinical pf malaria prevalence by qPCR.

**Community engagement**

A team of between 4 and 8 people depending on the size of the village and local resources, previously trained in community mobilisation activities, will be based at the village during 1-2 months preceding the first round of TCE and will remain in the village throughout the 12 months duration of the study. Specific community engagement activities will vary depending on the site’s population ethnicity and cultural and social background. The team will interact with the villagers at different levels to inform the community and engage it to participate in TCE implementation. Initial approval and support from the village leader with be sought. TCE purpose, procedures and expected risks and benefits will be explained as part of all community activities and meetings with villagers. These activities will include a diversity of events such as school sessions to engage children and their families, concerts, movies, plays and puppet shows performed on local holidays and written to convey information in an informal, entertaining way. Lunches and meals will be part of the events and will be designed to include teaching sessions. Focus group discussions (FGDs) will be conducted in groups of 8-12 representative individuals to discuss the purpose and benefits of TCE guided by community engagement trained staff. These activities will be all followed by Q&A sessions where specific questions can be raised by attending villagers of any age, which can then be used to modify the education activities according to the audience’s response. Quantitative and qualitative tools will be used to explore the understanding of the villagers of the project before and after the drug administration.

### 1.2 First exhaustive cross-sectional surveys and first TCE round

Exhaustive cross sectional surveys will be carried out in the selected villages (4 per site). Depending on the village size and level of participation, a total of about 3 days per village will be needed to conduct the first TCE round and associated survey. Individual informed consent will be asked from all participants or their attending relatives in individuals under 18 years of age (or lower depending on local guidelines). The samples (3 mL for participants 5.0 years and older, 0.5mls for those below 5 years) will be used for parasite DNA detection and gametocyte RNA assessment in those with >1000 copies/µL.

For each participant, in the TCE villages, blood sampling will be followed by a full treatment course of DP (total treatment of 3 days) plus PQ 0.25 mg/kg single dose. We will use the census data to follow-up and treat at home people who miss rounds of treatment; we will also follow-up at home any residents who miss cross-sectional surveys so that we can collect samples. When participants miss treatment or surveys due to being out of the village, messages will be left at home. Participants will be identified by biometrics (e.g. fingerprint or iris recognition), which will be linked to a unique identifier and barcode. Identification methods will be discussed per village. Possibilities include that individuals will receive a notebook (1 per family), a carton card or a bracelet with the barcode which can be used for later identification. All houses will be geo-localized by GPS.

### Second and third rounds of TCE and cross-sectional surveys

New rounds of 3 days treatment with DP+PQ will be administered to all participants of the 2 TCE villages at each site. Representative cross-sectional surveys will be carried out at monthly intervals.

-

### 1.4 Post-TCE survey

In all 4 study villages in each sites the exhaustive surveys will be repeated for detection of falciparum DNA and gametocytes as in the pre-TCE survey (see 2.1 below)

### 1.5 Follow up

Follow up period in all villages; all cases of clinical malaria in the residents as well as in the newcomers or visitors will be documented treated with a full course of DP. Throughout the study, the selected villages will be monitored closely. In both types of villages (TCE and non-TCE control) all inhabitants will be identified and given a unique ID number, and all houses will be geo-localized by GPS. The health posts’ activities will be closely supervised and malaria cases detected will be documented. Departures and arrivals of people will be documented. The community engagement teams will visit the villagers frequently and closely collaborate with the village leaders to strengthen participation. If a villager misses a TCE round or survey, then the study team will make attempts to follow up the person at home and by phone. If these attempts are not successful, a message will be left with the family head so that the team is informed when they return. People who leave the village for an extended time will be administered a catch up treatment or surveyed when they next become available. If newcomers arrive between surveys, they will be offered the option to become part of the study for as long as they are residents in an area where TCE is implemented, and also in the control villages newcomers will be identified, consented and surveyed.

### 1.6 Second year

In year 2 the control villages will receive TCE while the year 1 TCE villages will be observed. However this will be reviewed based on the results of the first year. A decision on the need to conduct TCE during a second year will be based on the prevalence measures at month 12 in the TCE villages. More sites in areas of known artemisinin resistance will be added in subsequent years should TCE prove successful in reducing malaria prevalence by ≥95% from baseline levels.

## Type and timing of surveys

There will be two types of surveys conducted throughout this study. There will be exhaustive cross-sectional surveys every 3 months on all village residents who consent to be surveyed (including those not enrolled initially for TCE), and the second type of survey will be on a smaller number of random samples once monthly.

### 2.1 Cross sectional surveys

Cross sectional surveys will be conducted on the entire village population who consent to be surveyed (including those not enrolled initially for TCE) starting with the first TCE round, then one month after the final round of TCE and thereafter at 3 month intervals. The approximate timing of the cross sectional surveys will be:

- First survey with TCE first round (month 0)
- First post-TCE (month 3)
- Post-TCE month 6
- Post-TCE month 9
- Post-TCE month 12 (1 year)

### 2.2 Other surveys

Additional surveys will be conducted on all *P.falciparum* carriers (or if the species cannot be determined) at monthly intervals to assess parasite and gametocyte dynamics. These surveys will be conducted from month 2 onwards throughout the first year.

### 2.3 Sample processing

At each survey, tympanic temperature will be measured and a very brief history of recent illness and travel will be obtained from each individual. Blood samples will be obtained consisting of 3.0 mL venous blood from all participants > 5.0 years and older, 0.5 mls for anyone below five. The samples will be used to detect asexual parasites (blood smear, sensitive PCR), parasite population structure (Sequenom genotyping and sequencing), gametocytes (microscopy, mRNA), Full blood counts (Sysmex where available) and hematocrits (or hemoglobin). The samples will be stored in a cool box in the field and then transported within 12 hours to the local laboratory for processing. Packed red blood cells, plasma and buffy coat will be separated, frozen and stored. The packed red cells will be transported frozen to the molecular laboratory at MORU for sample processing (DNA extraction or RNA isolation, quantitative PCRs). Plasma will be transported frozen to MORU for sample processing (a) immunological assays including antibody responses to malarial antigens and b) C-reactive protein levels). All samples for molecular analyses will be taken, handled and processed according to the SOPs developed specifically for these studies. If feasible, a 5 ml blood sample will be collected from all study participants who test positive for P.f. malaria during the study; this is for whole genome sequencing of the parasite. This may not be possible in some settings.

Suspected malaria patients will be tested on site by RDT and treated immediately if positive. Visitors and migrant cases will be documented.

## Drugs

The pilot TCE programme will be implemented via directly observed therapy (DOT) administration of an ACT treatment consisting of dihydroartemisinin-piperaquine (DP) accompanied by single dose primaquine (PQ) DOT will be administered at the health post.

One DP tablet contains 40 mg of dihydroartemisinin and 320 mg piperaquine. A weight-based regimen containing a total dose of approximately 7 mg/kg dihydroartemisinin and 55 mg/kg piperaquine phosphate will be used.

PQ 0.25 mg/kg single dose will be administered on day 1 of the 3 days DP regimen

## TCE protocol

All participants who have provided informed consent in the TCE villages will be given directly observed therapy (DOT) with DP for 3 days (target total dose of approximately 7 mg/kg dihydroartemisinin and 55 mg/kg piperaquine) plus low dose PQ (0.25 mg/kg, given on day 1 of DP). The drugs will be taken under supervision of the study team. These treatments are part of the National Treatment Guidelines in all countries where TCE will be implemented. The same procedure will be repeated monthly for 3 months. Thus, a total of 3 TCE treatments given in months 1, 2 and 3 will result in 3 rounds of TCE per year of study. Access to diagnosis and treatment will be similar for the paired TCE and non-TCE villages. A health post with RDTs and ACT+PQ for treatment will be established and regularly visited and re-supplied. Male and female villagers aged older than 6 months are the target study population and must meet the following inclusion and exclusion criteria.

### Inclusion criteria

- Age ≥6 months, male or female,
- Written informed consent (by parent/guardian in case of children)

### Exclusion criteria

- Pregnant women will not receive primaquine (urine pregnancy tests will be performed on women of appropriate age groups before drug administration at each TCE round)
- History of allergy or known contraindication to artemisinins, piperaquine or PQ
- Those who are, in the opinion of the study clinician, ill at the time of drug administration

### Withdrawal criteria

Each participant has the right to withdraw from the study at any time. In addition, the investigator may withdraw a participant if he/she considers it necessary for any reason including:

- Significant non-compliance with treatment regimen or study requirements
- Inablility to tolerate the medications i.e. and AE or SAE that necessitates withdrawal
- Loss to follow up

### Informed consent and eligibility

Participants will be screened by the study team to assess eligibility. Basic demographic and epidemiological data will be recorded by the study staff. Physical examination will be conducted by a qualified investigator. Weight and temperature will be recorded. All prescribed or over-the-counter and traditional medications used within the last 7 days will be recorded. Any drug allergies will be recorded. Screening tests will be performed as parasite counts from Giemsa or Field stained thick and thin blood films or malaria RDT, full blood count or haematocrit or Hb (depending on local resources) and urine pregnancy test for females of child bearing potential.

## Evaluation of participants’ perception, tolerability, safety and acceptability of TCE

The drugs used have been registered or evaluated extensively for malaria treatment but not as much as part of TCE. During the month 3 and the month 12 surveys a questionnaire will be administered to participants or their caregivers to evaluate perception, acceptability, tolerability and safety. Acceptability will be assessed both in terms of drugs received and possible side-effects as well as surveys requiring blood samples. Questions will specifically address whether individuals who refuse to participate or withdraw within the first three months because of unwillingness to give blood samples for the surveys would have joined the study (or not withdrawn) if it had involved receiving TCE without the need to give blood. Focus group discussions (FGDs) as well as in-depth interviews (IDIs) will be conducted to evaluate acceptability. IDIs will include the village leader as well as representative men, women and care givers per village. FGDs and IDIs will be moderated by trained staff based at the village.

The drugs used have been registered or evaluated extensively for malaria treatment but not as much as part of TCE. The months 3 and month 12 surveys questionnaires administered to participants or their caregivers will evaluate safety and will document adverse events (AEs). In this study, an AE is either a drug-related side effect or a new or exacerbation of a pre-existing symptom, sign or illness (other than malaria). A serious AE is one that results in death or is life-threatening, requires inpatient hospitalisation or prolongation of existing hospitalization. All SAEs must be reported by the site investigator to the Medical Monitor or his designee, within one day of his or her awareness of the SAE, who will then report to the study Data Safety Monitoring Board (DSMB). The site investigator must also report the SAEs to the local ethics committee in accordance with local requirements.

In a subsample of up to 10% of the study population a electrocardiogram will be obtained before and after the drug administration to document the safety of the study medication.

# VI. QUALITY CONTROL AND ASSURANCE PROCEDURES

The study will be conducted in accordance with the current approved protocol, ICH GCP, any national regulations that may apply to this study and SOPs. The MORU Clinical Trials Support Group (CTSG) will monitor adherence to SOPs for collection of clinical data and laboratory specimens and will perform quality checks (curation) of clinical and laboratory data according to standard methodologies. Malaria slide QC will be performed by MORU. Study sites may have in place a system for internal monitoring. In addition, regular external monitoring of all sites will be performed by the MORU CTSG according to a Monitoring Plan. Data will be evaluated for compliance with the protocol and accuracy in relation to source documents. The study drugs comply with regulatory requirements. The quality of study drugs will furthermore be assured by pharmacologic testing of a sample of study drugs in each site.

# VII. ETHICS

The Investigators will ensure that this study is conducted in compliance with the current revision of the Declaration of Helsinki 2013 and according to any National Regulations. The study protocol and its associated documents will be submitted to the Oxford Tropical Research Ethics Committee (OXTREC) and the appropriate local ethics committees for written approval. The Investigator will submit and, where necessary, obtain approval from the above parties for all substantial amendments to the original approved documents.

This study will use drugs that have been studied thoroughly and their toxicities are well described. In general, they are all well tolerated. The main reported effects for DP are gastrointestinal upset (nausea, vomiting, abdominal pain and diarrhoea) as well as dizziness, headache and disturbed sleep. Rates of these side effects are generally < 10% and often <5% except for dizziness (~12%) [11]. The main adverse effects for PQ are also gastrointestinal, and haemolytic episodes reported in G6PD deficient individuals. These effects however are not expected in this study, as the dose of PQ used will be lower (0.25 mg/kg) than the currently used (0.75 mg/kg) when administered as single dose for falciparum malaria treatment. Based on a PQ safety review recently conducted by MORU, the WHO MPAC modified its recommendation for PQ single dose falciparum treatment changing it from 0.75 mg/kg to 0.25 mg/kg in areas where the prevalence of G6PD deficiency is considered high enough to impose a risk to this population when G6PD testing is not available; this change eliminates the need for G6PD testing [10]. Primaquine will not be used in Cambodia as it is not in the local treatment guidelines.

# VIII. DATA HANDLING, ACCESS AND PUBLICATIONS

Direct access will be granted to authorised representatives from the sponsor and host institution and the regulatory authorities, if applicable, to permit trial-related monitoring and inspections. All study data will be recorded on standard Case Report Forms (CRF) and entered into a secure database. The participants will be identified by a study-specific participant number and/or code in any database. The name and any other identifying detail will NOT be included in any study data electronic file. Data may be used alone or in combination with data from related studies in secondary analyses. Any data published in the peer-reviewed medical literature will protect the identity of the subjects. All those who have made a substantial contribution will be coauthors on publications which will be open access. The sites have the right to publish their data individually and to include members of the sponsor’s team who have made a significant contribution. There will also be publications of pooled data which will be coordinated by the MORU group. All sites will have the opportunity to contribute to these publications.

# IX. REFERENCES

1. World Health Organisation (2011). .Global Plan for Artemisinin Resistance Containment. Geneva

2. Dondorp AM, Nosten F, Yi P, Das D, Phyo AP, et al. (2009) Artemisinin resistance in Plasmodium falciparum malaria. N Engl J Med 361: 455-467.

3. Phyo AP, Nkhoma S, Stepniewska K, Ashley EA, Nair S, et al. (2012) Emergence of artemisinin-resistant malaria on the western border of Thailand: a longitudinal study. Lancet 379: 1960-1966.

4. Hien TT, Thuy-Nhien NT, Phu NH, Boni MF, Thanh NV, et al. (2012) In vivo susceptibility of Plasmodium falciparum to artesunate in Binh Phuoc Province, Vietnam. Malar J 11: 355.

5. Amaratunga C, Sreng S, Suon S, Phelps ES, Stepniewska K, et al. (2012) Artemisinin-resistant Plasmodium falciparum in Pursat province, western Cambodia: a parasite clearance rate study. Lancet Infect Dis 12: 851-858.

6. Tran TH, Dolecek C, Pham PM, Nguyen TD, Nguyen TT, et al. (2004) Dihydroartemisinin-piperaquine against multidrug-resistant Plasmodium falciparum malaria in Vietnam: randomised clinical trial. Lancet 363: 18-22.

7. Smithuis F, Kyaw MK, Phe O, Aye KZ, Htet L, et al. (2006) Efficacy and effectiveness of dihydroartemisinin-piperaquine versus artesunate-mefloquine in falciparum malaria: an open-label randomised comparison. Lancet 367: 2075-2085.

8. Ashley EA, Krudsood S, Phaiphun L, Srivilairit S, McGready R, et al. (2004) Randomized, controlled dose-optimization studies of dihydroartemisinin-piperaquine for the treatment of uncomplicated multidrug-resistant falciparum malaria in Thailand. J Infect Dis 190: 1773-1782.

9. Ashley EA, McGready R, Hutagalung R, Phaiphun L, Slight T, et al. (2005) A randomized, controlled study of a simple, once-daily regimen of dihydroartemisinin-piperaquine for the treatment of uncomplicated, multidrug-resistant falciparum malaria. Clin Infect Dis 41: 425-432.

10. White NJ, Qiao LG, Qi G, Luzzatto L (2012) Rationale for recommending a lower dose of primaquine as a Plasmodium falciparum gametocytocide in populations where G6PD deficiency is common. Malar J 11: 418.

11. Myint HY, Ashley EA, Day NP, Nosten F, White NJ (2007) Efficacy and safety of dihydroartemisinin-piperaquine. Trans R Soc Trop Med Hyg 101: 858-866.
